# Clinical Knowledge and Practice of “Ketofol” at University of Gondar Comprehensive Specialized Hospital

**DOI:** 10.3389/fmed.2021.555973

**Published:** 2021-09-28

**Authors:** Nigist Alemayehu Woldekidan, Ammas Siraj Mohammed

**Affiliations:** ^1^Department of Clinical Pharmacy, School of Pharmacy, College of Medicine & Health Sciences, University of Gondar, Gondar, Ethiopia; ^2^Department of Clinical Pharmacy, School of Pharmacy, College of Health and Medical Sciences, Haramaya University, Harar, Ethiopia

**Keywords:** anesthesia, ketamine, propofol, practice, knowledge

## Abstract

**Background:** Ketamine and propofol in a single syringe are reported to create an admixture used for balancing cardiorespiratory effects during induction of general anesthesia. This study aimed to assess the clinical practice and knowledge of “ketofol” among anesthesia providers.

**Methods:** A cross-sectional institutional-based study was conducted among anesthesia providers. Data abstracted format was prepared and distributed to senior anesthetists, junior anesthetist postgraduate students, and undergraduate students. The study was conducted from January 1, 2019, to January 30, 2019. Descriptive statistics and binary logistic regression were performed for frequency distribution and to determine the association, respectively.

**Result:** From a total of 133 participants included in the study, the majority, 88 (66.2%), were men and 75 (56.9%) had 0–2 years of experience. More than two-thirds of participants, 105 (78.9%), have never had a seminar or educational session about combined use. Lack of experience among 11 participants (8.3%) was one of the reasons for not using “ketofol” followed by lack of knowledge among three (2.3%) participants. The majority of participants, 112 (84.2%), prefer ketamine and propofol to be administered one right after the other with separate syringes and the ratio to be 1:2, 64 (48.2). There was no significant association observed between sociodemographic and other characteristics and the practice of “ketofol.”

**Conclusion:** In this study, nearly half of the participants rated their knowledge at the average level, and the study identifies that there is clinical knowledge and practice gap among anesthesia providers working in the University of Gondar Comprehensive Specialized Hospital (UOGCSH). Preparing educational sessions regarding “ketofol” for addressing identified barriers is among the recommendations forwarded to UOGCSH.

## Introduction

Ketamine is a phencyclidine derivative, which is used as an intravenous anesthetic agent, and its mode of action is through causing dissociation anesthesia. Amnesic and analgesic effects, maintenance of muscle tone, protecting airway reflexes, and spontaneous respiration are among advantages that have been attributed to ketamine use. Nevertheless, different side effects were observed, including nausea, vomiting, raised blood pressure and heart rate, and development of hallucination, and also it was presumed to increase intracranial pressure (1, 2).

Propofol is neither opioid nor barbiturate, which is used for sedation and anesthesia providing rapid induction and recovery. Its mechanism of action involves the facilitation of inhibitory neurotransmission mediated by gamma-aminobutyric acid. Propofol has amnestic, anticonvulsant, and antiemetic properties. Its dose-related adverse effects include cardiovascular and respiratory depression (3–5).

Among the goals of procedural sedation and analgesia, the most common goals are achieving an adequate level of sedation with minimum pain and anxiety, increasing amnesia, decreasing drug-related adverse events, controlling behavior, and maintaining a stable cardiovascular and respiratory status procedural sedation and analgesia (PSA) (6). “Ketofol” has the advantage of reduced adverse effects of each drug counteracting the undesirable effects of one drug with the helpful effects of another. A combination of these drugs allows sedation to be achieved with a lower total dose of each drug, decreases the incidence of dose-related adverse effects, and ketamine can provide an analgesic effect that is absent when propofol is used alone. Because of this, physicians, nurses, and patients have shown a high level of satisfaction with its use (7, 8). This combination of safety and stability has also been proven (9).

A recent meta-analysis studied the safety and efficacy of ketofol in comparison with propofol as a single agent, which included six randomized control trials (RCTs), concluded that there was no statistically significant difference in safety. However, fewer respiratory events were documented in the combination group (10). Another meta-analysis by Jalili et al. (2016) corroborated the finding by revealing ketofol as a safe alternative to propofol for PSA (11). Moreover, the combination of ketamine and propofol appears to be safe in patients with cardiogenic shock, due to the lesser influence of ketamine on hemodynamic parameters (12). The recommended dosage is 0.5 mg/kg of a 1:1 mixture, followed by another dose of 0.5 mg/kg. A dose of 0.25 mg/kg is used afterward for maintenance of sedation (13).

As there is an increased use of this combination, the present study aims to assess the knowledge and practice of using “ketofol” admixture in clinical practice in the University of Gondar Comprehensive Specialized Hospital (UOGCSH). The second aim of this study is to assess whether age, sex, previous education, type of provider, and years of anesthetic experience affect their clinical practice and knowledge regarding “ketofol.”

## Method

### Study Area and Period

The study was conducted from January 1, 2019, to January 30, 2019, at the UOGSCH on anesthesia providers. UOGSCH is one of the oldest teaching hospitals in Ethiopia, which is located in northwest Ethiopia. The hospital provides service for more than 7 million people who come from Gondar town and surrounding district areas.

### Study Design

A hospital-based cross-sectional study was conducted on medical anesthesia fourth-year students and professionals who were practicing anesthesia at UOGCSH.

### Population

The source population includes professionals who are responsible for administering anesthesia, whereas the study was conducted on anesthesia providers who were on practice sites during the study period.

### Inclusion and Exclusion Criteria

All professionals who are responsible to administer anesthetic medication were included in the study whereas those who are not volunteered to participate in the study were excluded from the study.

### Sample Size Determination and Sampling Techniques

All consented health professionals who were administering anesthesia, from fourth-year students (they are allowed to administer anesthesia under close supervision) to senior anesthetists, were considered due to a small number of the study population, and a convenience sampling method was used to recruit the study participants.

### Study Variables

Clinical practice and knowledge about “ketofol” use were dependent variables whereas age, gender, work experience, and level of education were independent variables.

### Data Collection and Management

The data were collected by two investigators through a self-administered questionnaire. The data collection instrument was adapted from previous studies and consisted of sections focusing on Sociodemographic characteristics, the appropriate title of the participant, years of practicing anesthesia, and questions that assess the clinical knowledge and practice of ketamine-propofol (Ketofol) use. The data were collected from January 1, 2019, to January 30, 2019.

### Data Quality Assurance

A standard questioner was adapted from previous study and prepared to make fit with our setup (14). The questionnaire was pre-tested on 10 anesthesia practitioners of other close-related setups. The pre-tested data were not included in the final analysis, and some amendments were done to avoid any ambiguity before the beginning of actual data collection. The data were collected using a structured self-administered questionnaire. Additionally, the confidentiality of the information obtained was secured in such a way that the information will not be disclosed to anyone in anyway and will only be used for the study purpose. Each variable had been coded before data entry.

### Data Compilation and Analysis

After completing the data collection, the data were cleaned and checked for completeness. In addition, it was entered into the SPSS (IBM SPSS Statistics for Windows, Version 21.0; IBM Corp., Armonk, NY) for analysis. Sociodemographic and other baseline information were summarized using descriptive statistics, and categorical variables were expressed as frequencies and percentages. For the sake of analysis, knowledge level was categorized into low and high (average and high), while practice was categorized into poor and good, depending on predefined criteria. Binary logistic regression was performed to assess the factors associated with clinical practice and knowledge about “ketofol” use.

### Ethical Considerations

Ethical clearance was obtained from the ethics and research committee of the School of Pharmacy, the University of Gondar with the reference number of UoG-SoP/1164/2019. Informed oral consent was secured from the study participants prior to data collection. Information obtained from the study participants was kept confidential. Furthermore, participant identifiers were not used, and the data collected were used by the investigators only for the study.

## Result

### Characteristics of Participants

Among 150 participants, 140 responded to the questionnaire and among them seven were incomplete. The study population consist of 20 (15%) senior anesthetists, 25 (18.8%) postgraduate students, 6 (4.5%) junior anesthetists, and 82 (61.7%) undergraduate students.

Nearly half of the participants, 75 (56.4%), had 0–2 years of experience followed by 29 (21.8%) from 2 to 5 years, 25 (18.8%) from 5 to 10, 4 (3%) ≥10 years, and 88 (66.2%), the majority of the participants, of them were men (Table 1).

**Table 1:** Background characteristics of anesthesia provider at the University of Gondar Comprehensive Specialized Hospital (UOGCSH), Gondar, Ethiopia, 2019.

**Question**	**Response**	***N* (%)**
Age (year)	19–22	46 (34.6)
	23–26	54 (40.6)
	27–30	22 (16.5)
	31–34	8 (6.0)
	>35	3 (2.3)
Gender	Male	88 (66.2)
	Female	45 (33.8)
Select your appropriate title	Senior anesthetist	20 (15)
	Postgraduate student	25 (18.8)
	Junior anesthetist	6 (4.5)
	Undergraduate student	82 (61.7)
How many years have you been practicing anesthesia?	0–2years 2–5 years 5–10 years >10years	75 (56.4) 29 (21.8) 25 (18.8) 4 (3)

### Knowledge About “Ketofol”

A large number of participants, 116 (87.2%), have heard and used ketamine and propofol combination 113 (85%), and around half of the participants, 77 (57.9%), rated their knowledge at an average level (Table 2).

**Table 2:** Knowledge and practice of ketamine-propofol combination (ketofol) among anesthesia providers at the UOGCSH, Gondar, Ethiopia, 2019.

**Questions**	**Response**	***N* (%)**
Self-rated knowledge about “ketofol”	Low level	9 (6.8)
	Average level	77 (57.9)
	High level	47 (35.3)
Awareness about “ketofol”	Never heard about it	2 (1.5)
	Heard, but not used it	15 (11.3)
	Heard, and used it	116 (87.2)
Seminar or educational session about combination of ketamine-propofol	Yes	28 (21.1)
	No	105 (78.9)
Use of combination in the last 1 year at the same time	Yes	113 (85)
	No	20 (15)
Main reason for not using the combination	Lack of knowledge	3 (2.3)
	Lack of experience	11 (8.3)
	Lack of propofol	5 (0.8)
	Already used	113 (85)
Main advantage of “ketofol”	Hemodynamic stability	64 (48.1)
	To decrease adverse effects of occur when administered separately	51 (38.3)
	Effective analgesia	5 (3.76)
	To decrease dose of each	15 (11.3)
	For smooth induction	1 (0.75)
	Air way blunting	1 (0.75)
	I don't know	37 (27.82)
Indication to use “ketofol”	Stabilize vital sign	15 (11.3)
	Induction	47 (35.3)
	Sedation	4 (3)
	Analgesia	1 (0.75)
	I don't know	68 (51.13)
Preferred administration combination of ketamine and propofol at the same time	Mix them in the same syringe	10 (7.5)
	Administer one right after the other with separate syringes	112 (84.2)
	Administer in same time with separate IV line and syringes	2 (8.3)
Preferred dosage of ketamine and propofol that you administer	Ketamine 0.25 mg/kg and propofol 1 mg/kg	3 (2.3)
	Ketamine 1 mg/kg and propofol 2 mg/kg	42 (31.6)
	Ketamine 0.72 mg/kg and propofol 1 mg/kg	3 (2.3)
	Ketamine 0.5 mg/kg and Propofol 1 mg/kg	13 (9.8)
	Ketamine 2 mg/kg and propofol 1 mg/kg	8 (6)
	I don't know.	7 (5.3)
Preferred ketamine: propofol ratio that used, Respectively	1:1 ratio	57 (42.9)
	1:2 ratio	64 (48.2)
	2:1 ratio	12 (9)
Clinical practice that usually a combination of ketamine and propofol used	Induction	90 (67.7)
	Sedation	43 (32.3)
Rate the typical level of hemodynamic stability with the combination of ketamine and propofol when used as an induction agent	Very stable	26 (19.5)
	Stable	84 (63.2)
	Unstable	5 (3.8)
	Very unstable	2 (1.5)
	Sedation	16 (12)
Primary reason you have not used the combination as an induction agent	Lack of knowledge	56 (42.1)
	Lack of experience	49 (36.8)
	Time consuming to prepare	1 (0.8)
	Satisfied with the practice administering only one	11 (8.3)
	Sedation	16 (12)
The level of sedation/analgesia that most commonly “ketofol” used	Minimal (anxiolysis)	10 (7.5)
	Moderate (conscious)	58 (43.6)
	Deep sedation	62 (46.6)
	I haven't used it	3 (2.3)
Concerns with the use of the combination of ketamine and propofol	No concerns	28 (21.1)
	Concerns about compatibility of the two drug	60 (45.2)
	Uncertain about the benefit to using the two drugs together	22 (16.6)
	The combination is not approved by my department/institution	11 (8.4)
	The combination is not approved by FDA	14 (10.8)
	Hemodynamic status of patient	6 (4.5)
If a seminar or educational session regarding current knowledge and practice of the combination of ketamine and propofol was offered, how likely would you be to attend?	Very likely Somewhat likely Not at all likely	72 (54.1) 51 (38.3) 10 (7.5)

More than two-thirds of the participants, 105 (78.9%), have never had a seminar or educational session about the combination of ketamine-propofol, but in the last 1 year more than three-fourths of the participants, 113 (85%), have used ketamine-propofol combination at the same time. Those who did not use in the last 1 year cited that the main reason is lack of experience, 11 (8.3%), followed by lack of knowledge, 3 (2.3%), and lack of propofol, 5 (0.8%) (Table 2).

Regarding the question to list the main advantage of “ketofol”, the answer of the respondent involves for hemodynamic stability of 64 (48.1%), to decrease adverse effects to 51 (38.3%) that occur when administered separately, to decrease the dose of each 15 (113%). Almost half of the participants, 68 (51.13%), do not know about the indication of “ketofol” and nearly more than one-third, 47 (35.3%), put its indication as an induction agent (Table 2).

### The Preferred Ratio of Admixture

The majority of participants, 112 (84.2%), prefer ketamine and propofol to be administered one right after the other with separate syringes and the ratio to be 1:2, 64 (48.2%).

### “Ketofol” Usage for Sedation and Induction

More than one-third of participants, 90 (67.7%), used the combination of ketamine and propofol for induction and rate the typical level of hemodynamic stability for induction on stable, 84 (63.2%). Nearly half of the participants, 43 (32.3%), did not use the combination as an induction agent and put lack of knowledge as a primary reason followed by lack of experience, 49 (36.8%) (Table 2).

Around half of the participants, 62 (46.6%), use “ketofol” for deep and moderate sedation/analgesia, 58 (43.6%), and 60 (45.2%) of participants have worries about the compatibility of the two drugs and are uncertain about the benefit of using the two drugs together, 22 (16.6%).

Around three-fourth of participants, 72 (54.1%), are very likely to attend if a seminar or educational session regarding current knowledge and practice of a combination of ketamine-propofol was offered (Table 2).

Binary logistic regression was performed to identify the association of sociodemographic and other characteristics with the practice of “ketofol.” We have assessed the association between the sociodemographic and other characteristics, and the practices including the preferred ratio of administration (1:1 vs. others), a clinical practice that is usually a combination of ketamine and propofol used (induction vs. sedation), and preferred administration combination (mixing in the same syringe vs. other administration). However, each of the dependent variables has no significant association with the sociodemographic and other characteristics.

## Discussion

A combination of ketamine and propofol (Ketofol) has been proven to be effective in the operating room, ambulatory setting, and emergency department mixed in the same syringe (15–18).

A remarkable number of 116 (87.2%) of the respondents has used this combination drug and rated their level of knowledge about “ketofol” at an average of 77 (57.9%). Few numbers, 3 (2.3%), of participants, have raised lack of knowledge as a reason for not using combination drugs, but it was the main reason for most of the participants in the study conducted in the USA (14).

Most of the respondents, 112 (84.2%), prefer administration of ketamine and propofol to be administered one right after the other with separate syringes and with the ratio of 1:2, 64 (48.2%). Preference of mixing was significantly associated with the year of experience (*p* = 0.003) and the level of study of a professional (*p* = 0.004). In the previous study, single-syringe “ketofol” in a 1:1 ratio for sedation and analgesia of adults in the emergency department resulted in quick recovery, few adverse events, and patient and staff satisfaction (18).

In our study, there was no significant association observed between the year of experience and clinical use of “ketofol.” However, in another study, the use of “ketofol” for both sedation and anesthesia was associated with years of experience (7, 19).

Most of the respondents, 84 (63.2%), rate the level of hemodynamic stability as stable when the combination of ketamine and propofol is used as an induction agent. Even if the emergence of delirium is less prevalent, hemodynamic stability may be further required than the worrisome consequence of the condition (7, 18–20).

Almost half of the participants, 72 (54.1%), are very likely to attend a seminar or educational sessions regarding current knowledge and practice of the combination of ketamine and propofol. Similarly, the study in Minnesota, USA, shows the education had a statistical impact on the concern regarding the admixture. Also, web-based and face-to-face education have proven to be effective if required or mandatory. These factors of education have been studied in both nursing and other medical professions, with similar conclusions (14).

## Conclusion

This is the first attempt to assess clinical knowledge and practice of “ketofol” in UOGCSH. A large number of participants prefer ketamine and propofol to be administered one right after the other with separate syringes and the ratio to be 1:2, and nearly half of the participants rated their level of knowledge as average. Preference for mixing was significantly associated with the year of experience and the level of study of a professional. This study identifies clinical practice and knowledge differences among anesthesia providers of UOGCSH. As it is a critical area of patient care, the institution should prepare educational sessions to promote evidence-based medicine so that practitioners will update themselves by reviewing articles regarding “ketofol” admixture for the best care of the patient.

## Data Availability Statement

The original contributions presented in the study are included in the article/supplementary material, further inquiries can be directed to the corresponding author/s.

## Ethics Statement

The studies involving human participants were reviewed and approved by Ethics and research committee of School of Pharmacy and the Clinical Directorate of University of Gondar Comprehensive Specialized Hospital. Written informed consent for participation was not required for this study in accordance with the national legislation and the institutional requirements.

## Author Contributions

All authors listed have made a substantial, direct and intellectual contribution to the work, and approved it for publication.

## Conflict of Interest

The authors declare that the research was conducted in the absence of any commercial or financial relationships that could be construed as a potential conflict of interest.

## Publisher's Note

All claims expressed in this article are solely those of the authors and do not necessarily represent those of their affiliated organizations, or those of the publisher, the editors and the reviewers. Any product that may be evaluated in this article, or claim that may be made by its manufacturer, is not guaranteed or endorsed by the publisher.

## Abbreviations

FDA: Food and Drug Administration
UOGCSH: University of Gondar Comprehensive Specialized Hospital
USA: United States of America.

## References

[1] Green SM Krauss B. The semantics of ketamine. Ann Emerg Med. (2000) 36:480–2. 10.1067/mem.2000.109510 11054203

[2] Strayer RJ Nelson LS. Adverse events associated with ketamine for procedural sedation in adults. Am J Emerg Med. (2008) 26:985–1028. 10.1016/j.ajem.2007.12.005 19091264

[3] Claeys MA Gepts E Camu F. Haemodynamic changes during anaesthesia induced and maintained with propofol. Br J Anaesth. (1988) 60:3–9. 10.1093/bja/60.1.3 3257393

[4] Bahn EL Holt KR. Procedural sedation and analgesia: a review and new concepts. Emerg Med Clin North Am. (2005) 23:503–17. 10.1016/j.emc.2004.12.013 15829394

[5] Mirrakhimov AE Voore P Halytskyy O Khan M Ali AM. Propofol Infusion Syndrome in Adults: A Clinical Update. Crit Care Res Pract. (2015) 2015:10. 10.1155/2015/260385 25954513 PMC4410753

[6] Kliegman R Stanton B Behrman RE St. Geme JW, Schor NF, Nelson WE. Nelson textbook of pediatrics. (2016) 2.

[7] Willman EV Andolfatto G A. prospective evaluation of ketofol (ketamine/propofol combination) for procedural sedation and analgesia in the emergency department. Ann Emerg Med. (2007) 49:23–30. 10.1016/j.annemergmed.2006.08.002 17059854

[8] Alletag MJ Auerbach MA Baum CR. Ketamine, propofol, and ketofol use for pediatric sedation. Pediatric Emergency Care. (2012) 28:1391–5. 10.1097/PEC.0b013e318276fde2 23222112

[9] Friedberg B. Propofol-ketamine technique: dissociative anesthesia for office surgery (A 5-year review of 1264 cases) *Aesthetic Plast Surg*. (1999) 23:70-5. 10.1007/s002669900245 10022941

[10] Yan JW McLeod SL Iansavitchene A. Ketamine-propofol versus propofol alone for procedural sedation in the emergency department: a systematic review and meta-analysis. Acad Emerg Med. (2015) 22:1003–13. 10.1111/acem.12737 26292077

[11] Jalili M Bahreini M Doosti-Irani A. Ketamine-propofol combination (ketofol) vs propofol for procedural sedation and analgesia: systematic review and meta-analysis. Am J Emerg Med. (2016) 34:558–69. 10.1016/j.ajem.2015.12.074 26809929

[12] Li L Vlisides PE. Ketamine: 50 years of modulating the mind. Front Hum Neurosci. (2016). 10:612. 10.3389/fnhum.2016.00612 PMC5126726 27965560

[13] Lamperti M. Adult procedural sedation: an update. Curr Opin Anaesthesiol. (2015) 28:662–7. 10.1097/ACO.0000000000000244 26356290

[14] Olson AN Rao WR Marienau ME Smischney NJ. Period prevalence of ketamine-propofol admixture ketofol in the operating room among anesthesia providers at an academic medical center. Med Sci Monit Int Med J Exp Clin Res. (2015) 21:1737–44. 10.12659/MSM.893944 26077108 PMC4482185

[15] Camu F Vanlersberghe C. Pharmacology of systemic analgesics. Best Pract Res Clin Anaesthesiol. (2002) 16:475–88. 10.1053/bean.2002.0262 12516886

[16] Frey K Sukhani R Pawlowski J Pappas AL Mikat-Stevens M Slogoff S. Propofol versus propofol-ketamine sedation for retrobulbar nerve block: comparison of sedation quality, intraocular pressure changes, and recovery profiles. Anesth Analg. (1999) 89:317–21. 10.1213/00000539-199908000-00013 10439740

[17] Mortero RF Clark LD Tolan MM Metz RJ Tsueda K Sheppard RA. The effects of small-dose ketamine on propofol sedation: respiration, postoperative mood, perception, cognition, and pain. Anesth Analg. (2001) 92:1465–9. 10.1097/00000539-200106000-00022 11375826

[18] Gallo de Moraes A Racedo Africano CJ Hoskote SS Reddy DRS Tedja R Thakur L. Ketamine and propofol combination (ketofol) for endotracheal intubations in critically ill patients: a case series. Am J Case Reports. (2015) 16:81–6. 10.12659/AJCR.892424 25676819 PMC4332295

[19] Smischney NJ Beach ML Loftus RW Dodds TM Koff MD. Ketamine/propofol admixture (ketofol) is associated with improved hemodynamics as an induction agent: a randomized, controlled trial. J Trauma Acute Care Surg. (2012) 73:94–101. 10.1097/TA.0b013e318250cdb8 22743378

[20] Andolfatto G Willman E A. prospective case series of single-syringe ketamine-propofol (Ketofol) for emergency department procedural sedation and analgesia in adults. Academic Emergency Med. (2011) 18:237–45. 10.1111/j.1553-2712.2011.01010.x 21401785

